# Thermal imaging ruled out as a supplementary assessment in patients with fibromyalgia: A cross-sectional study

**DOI:** 10.1371/journal.pone.0253281

**Published:** 2021-06-16

**Authors:** Nuria Sempere-Rubio, Marta Aguilar-Rodríguez, Marta Inglés, Ruth Izquierdo-Alventosa, Pilar Serra-Añó

**Affiliations:** 1 Department of Physiotherapy, University of Valencia, Valencia, Spain; 2 Faculty of Physiotherapy, Department of Physiotherapy, UBIC Research Group, University of Valencia, Valencia, Spain; University of Würzburg, GERMANY

## Abstract

**Background:**

The diagnosis of fibromyalgia syndrome (FMS) syndrome is often complicated and relies on diagnostic criteria based mostly on the symptoms reported by patients. Implementing objective complementary tests would be desirable to better characterize this population.

**Objective:**

The purpose of this cross-sectional study was to compare the skin temperature at rest using thermography in women with FMS and healthy women.

**Methods:**

Eighty-six women with FMS and 92 healthy controls volunteered to participate. The temperature of all participants was measured by infra-red thermography, registering the skin surface temperature (minimum, maximum and average) at rest in different areas: neck, upper and lower back, chest, knees and elbows. In order to analyze the differences in the skin temperature between groups, inferential analyses of the data were performed using Mann-Whitney U test.

**Results:**

The results showed no significant difference in skin temperature between groups in the neck, upper back, chest and elbows (p>0.05). The lower back and knees areas showed significant differences between groups (p<0.05), although these differences did not reach a minimum of clinically detectable change.

**Conclusions:**

Women with fibromyalgia presented no clinically meaningful reduction or difference in skin temperature at rest when compared with a group of healthy women. The infra-red thermography is not an effective supplementary assessment tool in women with fibromyalgia.

## Introduction

Fibromyalgia (FM) is a common chronic pain syndrome accompanied by other symptoms such as fatigue, headache, sleep disturbances and anxiety [1,2]. This population show increased inflammation expression [3], due to a mitochondrial dysfunction-dependent event implicated in their pathophysiology [4]. Indeed, previous studies have reported high levels of the pro-inflammatory cytokines interleukin (IL)-6, IL-8 in peripheral blood [5,6].

Further, some previous studies have demonstrated that women with FM may have a distorted activity of the autonomous nervous system (ANS), showing alterations in the heart rate variability [7], sleeps disorders [8] and dysfunction of the microcirculation, affecting sweating functions and also body temperature [9,10].

FM is diagnosed according to the classification criteria established by the American College of Rheumatology (ACR) [11]. The diagnosis is not easy and may be frequently unattended [12]. The use of objective assessments to help with the diagnosis is essential to better characterize the syndrome and plan the therapeutic approach of this population [13].

Thermal imaging has been proved as an effective tool to study diseases in which skin temperature can reflect the presence of inflammation in underlying tissues due to a clinical abnormality in chronic pain conditions, such as in rheumatoid arthritis [14], osteoarthritis [15], frozen shoulder [16], and tendinitis [17]. This is because infrared radiation emitted by the human skin, reflects the real-time microcirculatory dynamics of the cutaneous surface of patients [18], being conditioned by the underneath metabolic activity, skin microcirculation and ANS activity [19]. The latter is well correlated with skin temperature [20] because of its role in human thermoregulation [21] and is also related with the exacerbation of the pain in FM population [22].

Pain is detected by primary afferent C-fibers that release substance P into the spinal cord, causing hyperalgesia and cooling. These findings suggest a relationship between the measurement of skin temperature and pain assessment. Therefore, this may be a good option in FM population because thermography is conducted with a portable camera that requires no contact with the individual [23], is non-ionizing [24] and non-invasive [25].

Nevertheless, there is no consensus about the usefulness of thermography. Some authors reported a greater number of hot spots in FM compared with healthy individuals [26,27] whilst others reported that the heat distribution patterns of people with FM are similar to those of the healthy population [28].

We hypothesized that the skin temperature in FM population is lower than in healthy population because of ANS impairment. Therefore, the goal of this study was to compare the skin temperature at rest between women with FM and healthy women.

## Materials and methods

### Participants and setting

A cross-sectional design was carried out. For the recruitment of the participants, a non-probabilistic sampling was performed. Specifically, a combination of an incidental sampling and a snowball-type sampling. First, women with FMS and their age-matched controls who met their respective inclusion criteria were contacted. Women with FM were recruited from several associations of FM and specialized medical units while healthy women were recruited through advertisements in local associations and on social networks. Further, once these participants were included as potential participants, recruitment continued through snowball sampling (i.e., word of mouth). They were recruited over a period of one year and six months. Of the total number of volunteers (i.e. 95 women with FM and 95 healthy controls), we discarded those who were not at the postmenopausal stage because of the well-known influence of the menstrual cycle on the temperature [29]. Therefore, a total of 86 women with fibromyalgia syndrome (FM) from different Fibromyalgia associations, and who met the diagnostic criteria of the American College of Rheumatology (ACR, 2010), as assessed by rheumatologists, formed the Fibromyalgia group (FMG) [11]. Ninety-two women, age-matched and without FM symptoms formed the control group (CG). Exclusion criteria for both groups were: suffering any rheumatologic, acute or terminal disease and failing to accomplish the requirements of the thermography measurement protocol recommended by Delphi study conducted by Moreira et al [23]. Further, none of them should present any symptom of inflammation or any sign of hot flashes due to the menopause.

All the assessments were carried out in the biomechanics laboratory of the Department of Physiotherapy at the University of Valencia.

This study complied with the ethical principles of the Declaration of Helsinki, adopted by the World Medical Association on medical research in humans and its revision in 2013. The project was approved by the Ethics Committee on Human Research of our institution. All enrolled participants provided informed written consent prior to the study.

Patients were not involved in the design, conduct, reporting, or dissemination of our research.

### Measurements

The participants’ skin temperature measurements were taken at the research laboratory, always at between 3 and 6 pm in an 8m^2^ room with black walls. The temperature was kept at 24° C and relative humidity at 44%, tested by a digital hygrometer (ThermoPro TP65), without any direct air source in the exploration area. The participants removed their clothes and remained standing in their underwear to acclimate to the room temperature. Body hair was removed from the exploration area. After 15 minutes, the skin temperature was measured [30] by a physiotherapist widely experienced in the analysis of skin surface temperature with a thermographic camera, using the standard protocol recommended by Gomes Moreira et al. in their Delphi study [23]. The participants were instructed to avoid alcohol and caffeine beverages, smoking, using cosmetics and showering for four hours before the assessment. They should have further avoided performing physical activity, receiving massage, physical therapy, saunas, electro diagnostic tests or taking steroids, sympathetic blockers, vasoactive drugs, opioids and transdermal patches, in the 24 hours prior to measurement. Participants were reminded of these recommendations by phone 48 h before the tests started.

The thermography camera was a FLIR E60BX Thermal Imaging Camera, [FLIR Systems, Inc., Oregon, USA; 76800 Pixels (320 x 240)], featuring a 320 x 240 60Hz infrared detector with a 0.045°C thermal sensitivity and a -20 to 120°C (-4 to 248°F) temperature range. The camera was turned on 30 minutes prior to the test to allow sensor stabilization following the manufacturer’s guidelines.

**Fig 1:**
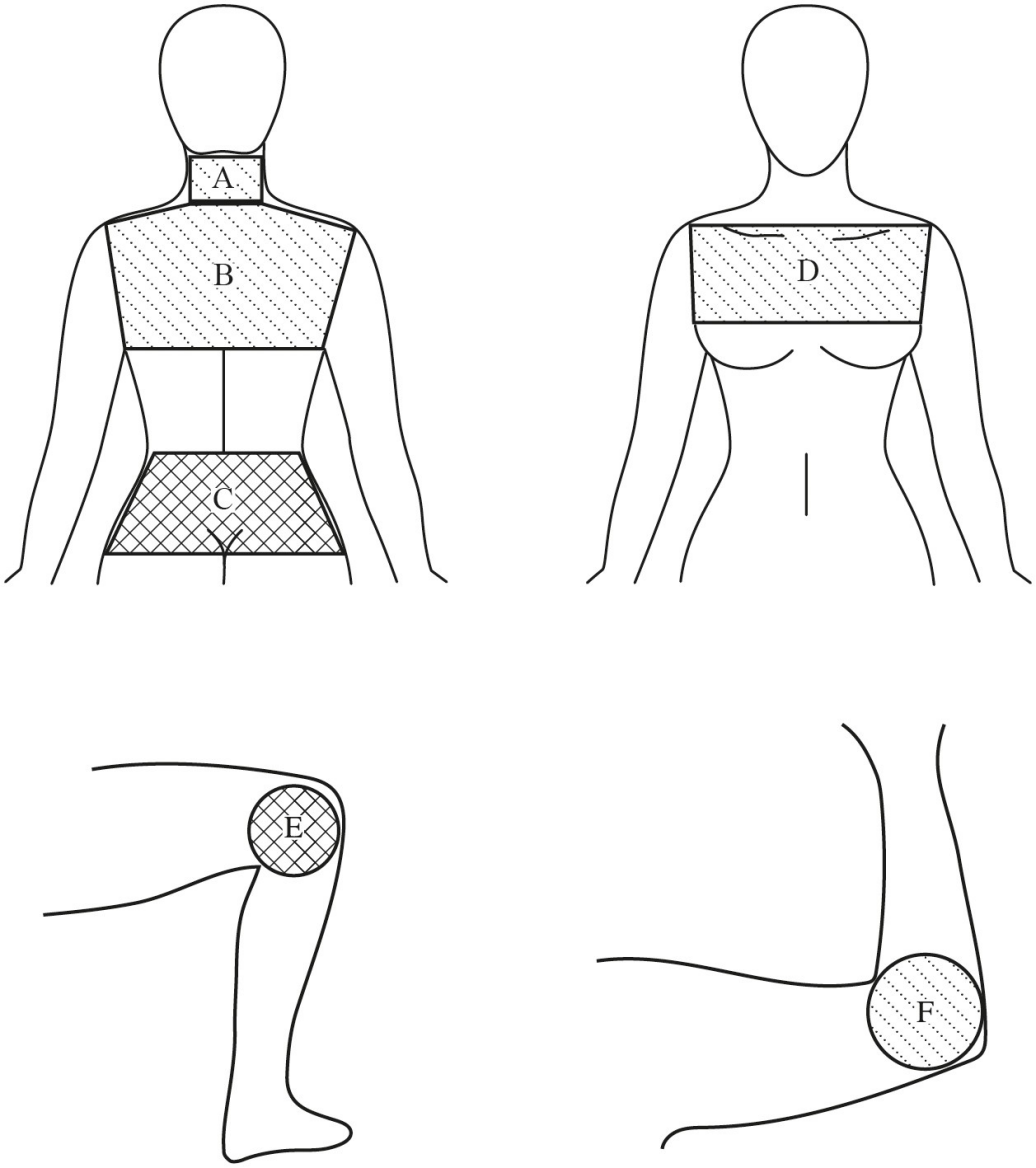
Regions of interest used in the study. A: Neck; B: Upper back; C: Lower back; D: Chest; E: Medial knee; F: Lateral elbow. Regions in which significant differences between groups were obtained are represented by bold lines.

The camera was perpendicularly pointed to the explored region using a tripod and at a distance of 1 m. The images were taken with an emissivity of 0.98 Ɛ [23,30]. The selected areas for our study were modified with regard to the Regions of Interest (ROI) described by Ammer et al. [31], in order to select the largest skin area possible. A total of six regions that are described below were assessed: neck, upper back, lower back, chest, knees and elbows (Fig 1). We discarded the assessment of more distal regions to avoid that the results could be influenced by other autonomic syndromes that have been described in people suffering from fibromyalgia, like Raynaud Syndrome [32] or peripheral sensory-motor polyneuropathy [33].

#### Neck

A rectangle was drawn on the back of the neck. The upper edge was adjacent to the hairline, while the lower edge corresponded to a horizontal line at the height of the spinous process of the first thoracic vertebrae. The lateral edges were the vertical outline of the neck.

#### Upper back

A polygon with six angles was drawn. The upper edge was a horizontal line at the height of the spinous process of the first thoracic vertebrae. From either end, two lines ran bilaterally to the upper lateral angle at the acromioclavicular joint. From this point, two lines ran bilaterally to the axillar fold. The lower edge was formed by the horizontal line joining these two latter points.

#### Lower back

A trapezius was drawn in which the upper edge was a horizontal line on the iliac crest. The lower edge was placed above the intergluteal cleft, adjacent to the natal cleft. The lateral edges were the outline of the skin.

#### Chest

A trapezius was drawn having an upper edge formed by a horizontal line connecting the two acromia. From either end two lines ran bilaterally to the axillar fold. The lower edge was formed by the horizontal line joining these two points. Medial knee. A circumference was drawn for each knee. The edge of the circumference was adjacent to the patella and the popliteal fold for its internal face. The average of the temperature values of the two knees were obtained for subsequent analyses.

#### Lateral elbow

A circumference was drawn for each elbow. The edge of the circumference was adjacent to the cubital fold and the lower edges of the elbow. The average of the temperature values of the two elbows were obtained for subsequent analyses.

The processing of the images was carried out using the *FLIR Research IR Professional Analyzing Software* (Version 1.2, Wilsonville, OR). A palette of 85–100 colors, with a thermal window of 0.15 uC for each color, was used. Thermal sensitivity of 0.51 uC was utilized per each shade, based on a rainbow-colored colorimetric scale, in which the colors ranged from the hottest (red) to the coldest (blue) [34]. The data obtained from each area were: the maximum, minimum and average temperature.

### Sample size calculation

Sample size was calculated taking into consideration a medium effect size of the mean difference of temperature between groups, a type I error of 5% and a type II error of 10%. This calculation resulted in 70 patients in each group. 86 women were included in the FMG and 92 in the CG. Ultimately, the temperature of the elbows and knees of 1 woman of the FMG could not be analyzed, including 85 women for the analysis of these regions. G-Power^®^ version 3.1. was used for sample size estimation (Institute for Experimental Psychology, University of Düsseldorf, Düsseldorf, Germany).

### Statistics

Statistical analysis was performed using SPSS software Version 22 (SPSS Inc., Chicago, IL, USA). Standard statistical methods were used to obtain the mean and standard deviation (SD). The normality assumption was checked using Shapiro-Wilk. Inferential analyses of the data were performed using Mann-Whitney U test with one inter-subject factor having two categories (FMG and CG).

As results of the Mann-Whitney U test, we provide the U statistic calculated as:

$$U=n_{1}n_{2}+\frac{n_{1}(n_{1}+1)}{2}-R_{1}$$

Where n_1_ is the sample size for the FMG and n_2_ is the sample size of CG and R_1_ is the sum of ranks for group with FM.

Further, we provide the Z-score to convert the statistic U in a standardize value. The effect size, when significant differences were obtained, was calculated using r (z/√N).

Furthermore, to explore the relationship between suffering the syndrome and the different categories of the demographic data (i.e. civil and employment status, studies level, smoking habits and alcohol intake), we conducted Chi Square tests (χ^2^). The type I error for the statistical tests was set at 5% (p ≤ 0.05).

## Results

### Participants

The mean (SD) age for CG was 54.89 (6.31) years whilst for FMG it was 54.91 (7.00) years. There was no significant difference in age between groups (p>0.05). The mean (SD) body mass index (BMI) for CG was 27.83 (4.75) whilst for FMG it was 25.97 (4.00). There was a significant difference in BMI between groups (p<0.05) being both values included in the ‘overweight’ category [35]. Table 1 shows demographic data of the participants.

**Table 1:** Demographic data of the participants.

	FM	C	p-value of the χ^2^
Civil Status			
Married/in union	68	66	χ^2^(3) = 3.10, p = 0.38
Single	6	11	
Widowed	1	4	
Separate/Divorced	11	11	
Employment status			
Active	44	60	χ^2^(3) = 4.31, p = 0.23
Unemployed	18	14	
Pensioner	21	14	
Housekeeper	3	4	
Studies level			
Illiterate	1	2	χ^2^(3) = 15.01, p < 0.05*
Primary	37	28	
Secondary	36	26	
University	12	36	
Smoking habits			
Daily	19	14	χ^2^(3) = 3.04, p = 0.39
Not daily	3	7	
Not at the present	31	30	
Never	33	41	
Alcohol intake			
Daily	6	5	χ^2^(4) = 3.26, p = 0.52
Some days per week	25	34	
Some days per month	15	21	
Some days in the last year	16	14	
Not in the last year	24	18	

FM: Fibromyalgia group; C: Control Group;

*: Statistical relationship among the categories (p<0.05).

### Thermography outcomes

Table 2 shows the results of the thermographic assessment. Our results showed that there were no significant differences in the skin temperature between groups in the neck, upper back and chest (p>0.05). There were significant differences between groups in the lower back for minimum temperature (U = 3210.00, Z = -2.17, p<0.05), maximum temperature (U = 3270.00, Z = -2.00, p<0.05) and average temperature (U = 3161.00, Z = -2.32, p<0.05). Further, there were significant differences between groups in the knees for minimum temperature (U = 3038.50, Z = -2.56, p<0.05), maximum temperature (U = 3124.00, Z = -2.31, p<0.05) and average temperature (U = 2738.50, Z = -3.44, p<0.05). There were not significant differences between groups in the elbows assessment for minimum temperature, maximum temperature and average temperature (p>0.05). The significant differences obtained did not exceed 0.5°C which is considered the minimum clinical finding (44) Fig 1 show the regions in which significant differences were obtained in bold lines.

**Table 2:** Skin temperature of the study participants.

	FM		C		Size effect (r)
	Mean (SD)	Median (range)	Mean (SD)	Median (range)	
**Neck_min**	31.58 (1.28)	31.60 (27.45–34.05)	31.89 (1.06)	31.93 (26.75–34.20)	
**Neck_max**	34.53 (0.93)	34.73 (30.30–36.10)	34.43 (1.00)	34.58 (29.95–36.25)	
**Neck_ave**	33.56 (1.03)	33.60 (29.35–35.50)	33.57 (0.98)	33.68 (28.80–35.45)	
**Upper back_min**	30.53 (1.26)	30.58 (26.65–33.50)	30.39 (1.32)	30.28 (26.25–33.15)	
**Upper back_max**	33.75 (0.99)	33.85 (29.90–35.60)	33.63 (1.04)	33.75 (29.50–35.65)	
**Upper back_ ave**	32.15 (1.09)	32.15 (28.20–34.45)	32.10 (1.18)	32.10 (27.95–34.35)	
**Lower back_min**	29.75 (1.44)	29.75 (26.60–32.45)	29.25 (1.42)*	29.20 (25.90–31.85)	0.16
**Lower back_max**	33.99 (1.08)	34.15 (30.55–35.85)	33.65 (1.13)*	33.78 (30.55–35.85)	0.15
**Lower back_ ave**	31.68 (1.25)	31.55 (28.30–34.20)	31.21 (1.28)*	31.25 (28–33.85)	0.17
**Chest_min**	30.82 (1.20)	30.88 (27.30–33.90)	30.64 (1.22)	30.65 (27.75–33.35)	
**Chest_max**	34.28 (0.77)	34.25 (32.55–36.05)	34.09 (0.91)	34.15 (32.30–35.95)	
**Chest_ ave**	32.34 (0.98)	32.28 (30.05–34.90)	32.19 (1.13)	32.15 (29.50–34.25)	
**Knee_min**	29.70 (1.12)	29.70 (27.35–32.25)	29.23 (1.03)*	29.28 (27.35–31.35)	0.19
**Knee_max**	31.86 (1.02)	31.83 (29.75–34.50)	31.49 (0.95)*	31.40 (29.70–34.65)	0.17
**Knee_ave**	30.80 (1.03)	30.75 (28.70–33.30)	30.21 (0.97)*	30.15 (28.35–32.25)	0.26
**Elbow_min**	30.28 (1.03)	30.45 (27.25–32.75)	30.05 (0.93)	30.15 (26.05–31.75)	
**Elbow_max**	32.79 (0.85)	32.80 (30.60–35.30)	32.61 (0.74)	32.70 (30.70–34.05)	
**Elbow_ave**	31.52 (0.89)	31.50 (29.15–33.95)	31.34 (0.80)	31.40 (29.70–33.15)	

FM: Fibromyalgia group; C: Control Group; min: Minimum temperature of the region; max: Maximum temperature of the region; ave: Average temperature of the region;

*: Statistical differences between groups (p<0.05).

## Discussion

The present study was designed to identify possible differences in the skin temperature, at rest, between women with FM and healthy women in order to consider thermal skin imaging as a supplementary assessment of FM. Thermal assessment at rest is a simple and noninvasive tool that can provide a reliable non-fatiguing assessment in this population. Since previous studies evidenced inflammation of the small muscle fibers in people with FM [36–39] some authors predicted that infrared thermography could be useful to detect peripheral temperature changes derived from peripheral inflammation, as has been shown for other pathologies [40–47]. Further, we explored the thermal patterns of the body at rest because of the need to dispose of non-fatiguing and easy-to-use assessments. We took into consideration that people with FM refer fatigue when exertion is required [48], so we decided to use an assessment in a non-fatiguing environment. The results derived from our study revealed no statistical or clinical differences in the body surface temperature between groups in most of the analyzed areas. The temperature of the lower back and knee in FM women was higher than that obtained in healthy women, but this difference, although statistically significant, did not exceed 0.5°, either in the average or maximum temperature, as required to be considered a clinical finding [49]. Therefore, this study demonstrates that, in a resting condition, infrared thermography detects no changes in body heat in women with FM due to possible alterations in the surface blood flow or alterations in the microcirculation of ANS and therefore this tool should not be used to assess ANS disorders in this syndrome [9].

Our results are not consistent with those derived from two previous studies, conducted by the same research group, in which thermography was used as an assessment tool to differentiate the thermal pattern in people with FM and their healthy controls [26,27]. Both studies analyzed whether there were hot spots in certain body areas in people with FM or myofascial pain, these hot spots being identified as differences in temperature of more than 0.5° with respect to the surrounding areas. Both studies reported that the FM group had a greater number of hot spots than the healthy group.

However, despite these positive results, thermography was considered as a specific but barely sensitive tool, although the exact sensitivity and specificity was not reported. However, such results are not exactly comparable with ours because, as noted, these studies used the fibromyalgia diagnosis criterion which involves 18 tender points [50], subsequently invalidated [1], unlike the approach used in our study, which explores the skin temperature pattern at rest. These studies measured the differences in the numbers of hot spots between people with and without FM. A hot spot is defined as a small area at least 5°C warmer than the surrounding area and therefore does not represent an increase in the temperature of larger areas included in the diagnostic criteria which are supposedly painful. We hypothesized that the skin temperature of this areas in FM population would be different than those of their healthy counter-parts because of ANS impairment. Broader areas are expected to be affected due to the complex relationship of the different autonomic structures of the neuraxis [51]. Previous studies assessing people with arthritic diseases have indicated significant differences in heat distribution indices compared to healthy people as reported by the review of Jasti *et al*. [52] Thus, our results are not completely comparable with those obtained from other previous studies.

In order to resolve the discrepancies between the results of previous studies on the usefulness of the tool, we designed a study ensuring a sufficient recruitment of participants to achieve a statistical power of 90% that would give greater reliability in the results than the previous studies, and therefore, the probability of committing a type II error or false negatives was low. Accordingly, the power of the study allows us to discard the hypothesis, since the thermal pattern between groups is similar.

Our results, therefore, suggest that there may be an equivalence of the local circulatory dynamics of the analyzed groups, while thermography fails to enable objectifying a reduction in the amount of blood in the areas evaluated in women with FM. This would be consistent with the results of the study by Dibai Filho’s group, in which they also found no temperature differences between women with chronic neck pain and healthy women and which already suggested that the circulatory dynamics would be similar in both groups without a reduction of blood in subjects with pain [53].

Previous studies suggest that pain in FMS is caused by central and peripheral sensitization [54], altered pain mechanisms and the presence of inflammatory substances both in the spinal cord, brain and the peripheral tissues [55]. However, it is possible that this peripheral inflammation (neuropeptides, chemokines and cytokines), reported in previous studies [3,6,56], is not revealed at the depth of 5 mm where infra-red radiation is emitted by the skin [57]. In this regard, we did not determine any peripheral biomarker of inflammation, since it was beyond the scope of the manuscript. Likewise, there are other trends suggesting that central sensitivity could play a more important role in the perception of pain in FMS [58], due to a reduced central inhibition of pain, which perpetuates it and broadens its nociceptive signal [59]. Thus, people with FM can refer pain without any type of stimulus, something which could explain why this study fails to report differences in temperature between healthy women and women with fibromyalgia at rest.

We need to take into consideration that the assessments were conducted at rest, since our goal was to explore the usefulness of this method as an easy-to-use complementary clinical technique to help in the assessment of the FM. Further studies analyzing the thermal response during sportive activities could provide interesting information regarding thermal patterns in this population.

Further, several study limitations need to be considered when evaluating our results. First, this is a cross-sectional study, so no cause-effect can be drawn from our results, since there were no follow-up of the participants. Second, people with FM conformed a heterogeneous population thus, even though the sample size calculation was well conducted and more participants than necessary were recruited, the heterogeneity may require even a larger sample size. Besides, a non-probabilistic sampling method of recruitment was used that may jeopardize the representativeness of the sample. Future studies using subgroups, exploring the effect of other possible confounder variables and performing other sportive or active protocols would provide more precise information.

## Conclusions

There are no clinical differences in skin temperature at rest between women with FM and healthy women in the core regions of the body except for the low-back region in which the significant differences did not achieve a minimum of clinically detectable change. Further, women with FM showed a significant higher temperature in knees although the differences also did not reach the minimum of clinical detectable change. Infra-red thermography is not an effective supplementary assessment aid in women with fibromyalgia.

## Supporting information

S1 Data (XLSX)
